# Complex Refractive Index Spectrum of CsPbBr_3_ Nanocrystals via the Effective Medium Approximation

**DOI:** 10.3390/nano15030181

**Published:** 2025-01-24

**Authors:** Sang-Hyuk Park, Jungwon Kim, Min Ju Kim, Min Woo Kim, Robert A. Taylor, Kwangseuk Kyhm

**Affiliations:** 1 Clarendon Laboratory, University of Oxford, Parks Road, Oxford OX1 3PU, UK; sanghyuk.park@physics.ox.ac.uk (S.-H.P.); robert.taylor@physics.ox.ac.uk (R.A.T.); 2 Department of Opto & Cogno Mechatronics Engineering, Research Center for Dielectric Advanced Matter Physic (RCDAMP), Pusan National University, Busan 46241, Republic of Korea

**Keywords:** complex refractive index, perovskite, colloidal quantum dots, effective medium approximation

## Abstract

We have estimated the intrinsic complex refractive index spectrum of a CsPbBr_3_ nanocrystal. With various dilute solutions of CsPbBr_3_ nanocrystals dissolved in toluene, effective refractive indices were measured at two different wavelengths using Michelson interferometry. Given the effective absorption spectrum of the solution, a full spectrum of the effective refractive index was also obtained through the Kramers–Krönig relations. Based on the Maxwell–Garnett model in the effective medium approximation, the real and imaginary spectrum of the complex refractive index was estimated for the CsPbBr_3_ nanocrystal, and the dominant inaccuracy was attributed to the size inhomogeneity.

## 1. Introduction

Recently, perovskite nanostructures have gained a lot of attention for their remarkable optoelectronic properties [1,2,3]. For example, large quantum yield, narrow emission spectrum, and wavelength tunability are very useful for various optoelectronic devices such as light-emitting diodes [4,5], solar cells [6,7,8], and sensors [9,10]. The remarkable progress of synthetic methods enables us to control the shape of CsPbX_3_ perovskite nanostructures such as nanocrystals (NCs) [11], nanowires [12], nanosheets [13], and nanoplatelets [14]. For precise optical manipulation of those nanostructures, both refractive index and absorption coefficient are necessary [15,16,17]. For example, the design of tandem cells [18], light-emitting diodes [15,17], laser [16,19], and waveguide [20] can be optimized, provided that the complex refractive index is given.

In the case of film structures, ellipsometry [21,22,23] is widely used to measure refractive index. As spectroscopic ellipsometry measures the polarization change of reflection light from the surface, non-uniform thickness and roughness can produce an inaccurate result due to sparkle scattering. Recently, advanced models were used to account for surface roughness [24] and ligand-induced packing fraction [25] in NC films. However, traditional ellipsometry often assumes non-interacting dilute particles for NC ensembles. This limits the accuracy, and interparticle interactions are considered to play a crucial role [26]. Therefore, high concentrations of NCs can further complicate the optical response, leading to difficulties in obtaining precise refractive index. Moreover, preparation methods and film inhomogeneity also hinder consistent data acquisition.

For solution-state nanoparticles, various approaches have been proposed for determining the refractive index based on the principles of scattering theory. For instance, the refractive index matching technique [27] requires specific solvents to match the refractive index, replacing solvents that can potentially affect the stability of the nanoparticles. Other methods that involve measuring scatted light from nanoparticles employing the integrating sphere [28], flow cytometry [29,30,31], and nanoparticle tracking analysis [32,33] have been suggested based on scattering theory. However, the accuracy of these approaches diminishes for non-spherical particles and particles smaller than 50 nm. Moreover, scattering-based methods can be inaccurate due to the influence of absorption and fluorescence of nanoparticles.

To overcome those limitations of the oversimplified assumption in spectroscopic ellipsometry and the experimental inaccuracy and the size-related constraints in from the scattering-based methods, the measurement of refractive index in colloidal solution was studied [34,35,36,37]. Based on the assumption of non-interacting particles, this method uses an iterative procedure using the Kramers–Krönig (KK) relation and absorption spectrum. When spectral integration is calculated for the KK relation, the finite spectrum range also gives rise to an additional inaccuracy. Although several methods were proposed [38,39,40,41], the inaccuracy of measurement and analysis was not discussed systematically. In this work, we propose a modified method to obtain a complex refractive index spectrum of a CsPbBr_3_ NC from the colloidal solution. For various concentrations of CsPbBr_3_ NC solutions, the effective refractive index was measured at two reference wavelengths using the Michelson interferometer, and the spectrum of effective refractive index was calibrated using the KK relations and absorption spectrum. Subsequently, the full spectrum of the complex refractive index of intrinsic CsPbBr_3_ NC was obtained based on the Maxwell–Garnett EMA.

## 2. Experiment

**Figure 1:**
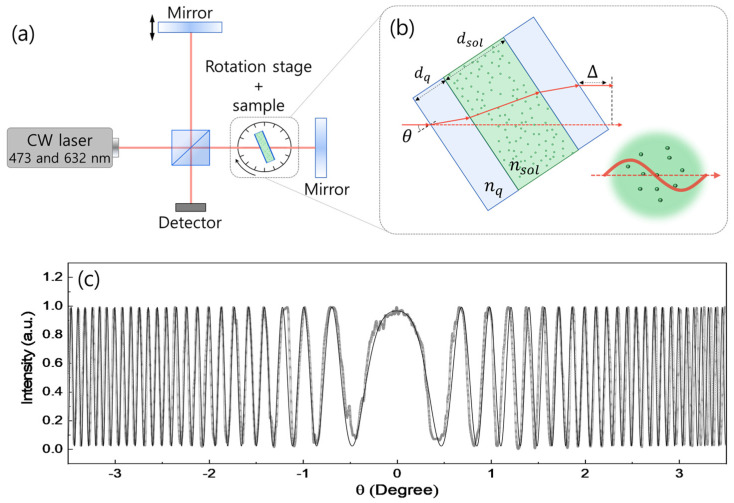
(**a**) Refractive index is measured with the Michelson interferometer with two continuous wave (CW) lasers (473 nm and 632 nm), where the intensity interference is measured for changing the incident angle of the laser to sample (*θ*). (**b**) Schematics show the laser beam path in the cuvette, which contains the solution. The light scattering with nanocrystals is considered in the Maxwell–Garnett approximation, where light wavelength needs to be far larger compared with the particle size. (**c**) Angle dependence of the laser interference at 632 nm is measured with 1.50 mg/mL concentration of CsPbBr_3_ nanocrystals in toluene (thick gray) and compared with a theoretical formula of Equation (1) (thin black).

For sample preparation, a commercial product of CsPbBr_3_ nanocrystals (Sigma Aldrich/product number 900746) was purchased and dispersed in toluene at various concentrations from 0.50 mg/mL up to 2.0 mg/mL. The CsPbBr_3_ nanocrystals were passivated with oleic acid and oleylamine, effectively minimizing the influence of intrinsic bromide vacancies on the refractive index measurements [42,43,44,45,46]. The dilute solutions were contained in a quartz cuvette and dropped and cast on a glass substrate separately for transmission electron microscopy (TEM). For the measurement of the refractive index, the Michelson interferometer was utilized, as shown schematically in Figure 1a. Given two monochromatic light sources of continuous wave (CW) lasers, the He-Ne laser with a wavelength of 632.8 nm and average power of ~943 µW, and a diode-pumped solid-state laser with a wavelength of 473.0 nm and average power of ~348 µW, two perpendicular paths are separated using a beam splitter. While the reflected one returns to the beam splitter using a mirror mounted on a translation stage, the transmitted one passes through a cuvette containing the NC solution, reflects off another fixed mirror, and subsequently returns to the beam splitter. Provided that the two beams are collimated well, a circular fringe pattern can be observed due to interference. The bright central fringe can be used for the optimum conditions and filtered by an iris before the detector. As the cuvette rotates, the optical path difference changes the central fringe intensity.

As shown schematically in Figure 1b, the rotating sample gives rise to different paths (solid in red) in the cuvette and air (Δ) compared to the optical path of normal incident (dotted in red). The refracted path with an incident angle *θ* is associated with the thickness and the refractive index of the cuvette (*d*_q_, *n*_q_) and solution (*d*_sol_, *n*_sol_), respectively. These enable us to formulate angle dependence of the intensity *I*(*θ*, *λ*) in interference at a certain wavelength *λ* as [47]:

(1) $$I\left(\theta \right)\propto cos\left[\begin{array}{c} \frac{2\pi }{\lambda }\left(4d_{q}\left(\sqrt{n_{q}^{2}-\sin^{2}\theta }-\cos\theta \right)+2d_{sol}\left(\sqrt{n_{sol}^{2}-\sin^{2}\theta }-\cos\theta \right)\right)\\ -2\times \left(2n_{q}d_{q}+n_{sol}d_{sol}-2d_{q}-d_{sol}\right) \end{array}\right].$$

Figure 1c shows one of our examples, where Equation (1) (thin black) was fitted to the experiment (thick gray) for a solution with 1.50 mg/mL concentration of NC measured at a wavelength of 632.8 nm. The thickness of the gray line represents technical inaccuracy, and the angle increases with a step of 0.1 degree.

As colloidal CsPbBr_3_ NCs are dispersed in toluene solvent, our interferometer provides an effective refractive index. Effective medium approximation (EMA) is widely used to estimate the effective refractive index of composite materials made up of multiple distinct phases. The EMA is particularly useful in situations where the particles dispersed in a host medium are much smaller than the wavelength of the interacting light. In this case, the particles can be treated as an optically homogeneous medium, whereby the overall effective refractive index of the solution is altered.

The Maxwell–Garnett (MG) [48] and Bruggeman [49] models are two of the commonly used EMA models for the prediction of the optical properties of composite materials. The Bruggeman model is suitable for materials with comparable volumetric contributions and interactions as it treats the components symmetrically. On the other hand, the Maxwell–Garnett model is suitable for host-guest systems with a low volume fraction of dispersed particles, where the embedded particles interact minimally within a continuous host. Therefore, the NC solution can be described by the MG model as the guest NCs dispersed in a host solvent that is far smaller than the light wavelength, as shown schematically in Figure 1b.

In relation to the orientation of NCs, this study assumes a random orientation distribution of the NCs within the colloidal solution. This random orientation is a typical characteristic of colloidal systems wherein the NCs possess the freedom to rotate and align in diverse directions, resulting in an averaged refractive index across the entire system. While some studies have taken into account the effect of nanoparticle alignment in the context of asymmetric nanoparticle shapes [37,50], the NCs used in this study have a symmetric cubic shape, and thus, the anisotropic effect of individual NC orientation is rendered negligible. Consequently, the present study focuses on providing a generalized model for random orientations, which is often more relevant for colloidal systems in practical applications.

## 3. Result and Analysis

The CsPbBr_3_ in orthorhombic perovskite crystal structures is composed of corner-sharing [PbBr_6_]^4−^ octahedra, where lead (Pb^2+^) resides at the center, surrounded by six bromine (Br^−^) ions. These octahedra form a three-dimensional framework, with cesium ions (Cs^+^) occupying the voids between them to maintain charge neutrality. In this orthorhombic structure, the Pb-Br-Pb bond angles deviate from 180°, leading to tilting and distortion of octahedra [51]. This tilting reduces the symmetry compared to the cubic phase at high temperatures [52], whereby the band gap energy and exciton levels are affected. The optical transitions responsible for absorption and emission primarily originate from the interaction between the Br (p orbital) and Pb (s orbital), leading to a direct band gap of 2.3~2.4 eV [53,54].

**Figure 2:**
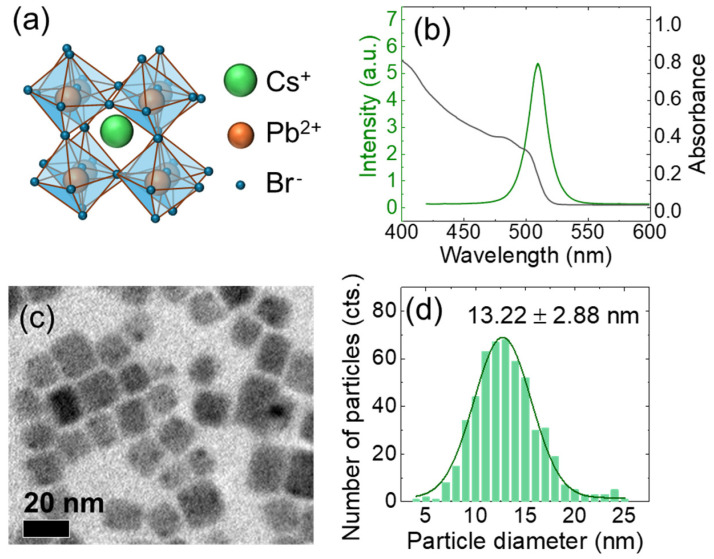
(**a**) Crystal structure of CsPbBr_3_ perovskite in orthorhombic phase at room temperature, showing Cs^+^ atoms (green), Pb^2+^ atoms (orange), and Br^−^ atoms (blue) is shown schematically. (**b**) Photoluminescence and absorbance spectrum of a solution of CsPbBr_3_ nanocrystals. (**c**,**d**) Given TEM image of dispersed CsPbBr_3_ nanocrystals, a histogram of size distribution is obtained and fitted with a Gaussian function.

In the case of CsPbBr_3_ NCs, the exciton energy level depends on their confinement size, and the size distribution gives rise to a spectral broadening [55,56,57,58]. In Figure 2b, photoluminescence (PL) and absorbance spectrum are shown in CsPbBr_3_ NCs dispersed in toluene. The PL peak appears at 510 nm with a linewidth of 20.3 nm under a 355 nm excitation, and a spectral Stokes shift of 14 nm can be obtained with respect to the absorbance shoulder at ~496 nm. Given the transmission electron microscopy (TEM) image of CsPbBr_3_ NCs in Figure 2c, a size distribution of the cubic NCs was obtained in Figure 2d. The average size of $\bar{L}=13.22$ nm was obtained with a standard deviation of 2.88 nm, which corresponds to 22% of size inhomogeneity. Therefore, the size-to-wavelength ratio of 473 nm and 632 nm meets the condition of the Maxwell–Garnett model (*L*/*λ* ≤ 0.03). Furthermore, since the average size of the NCs is much larger than the exciton Bohr radius (~7 nm for CsPbBr_3_) [59,60,61], quantum confinement effects are minimized, allowing intrinsic optical properties to be probed primarily without significant size-dependent changes.

**Figure 3:**
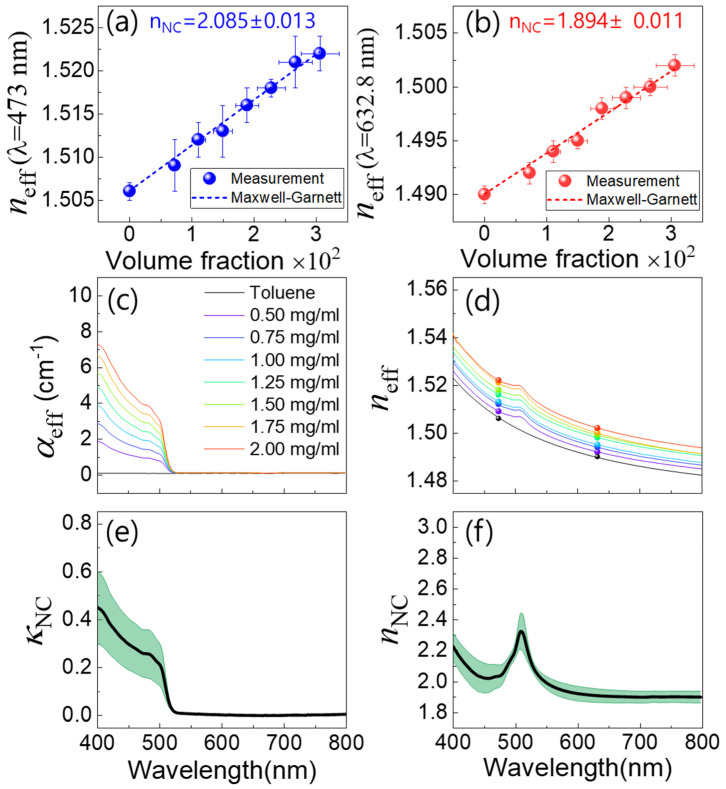
(**a**,**b**) Concentration dependence of the effective refractive index in CsPbBr_3_ (*n*_eff_) solution (*n*_eff_) measured at 473 nm and 632 nm are shown in terms of volume fraction, respectively. Those results are compared with the formula of Equation (2) in the Maxwell–Garnett approximation, where the average refractive index of CsPbBr_3_ nanocrystals (*n*_NC_) is used. (**c**,**d**) Given the spectrum of the effective absorption coefficient (*α*_eff_) in CsPbBr_3_ solution for various concentrations, the *n*_eff_ spectrum is also obtained using the Kramers–Krönig relation and the two reference refractive indices measured at 473 nm and 632 nm. The scatters in (**d**) correspond to the values shown in (**a**,**b**). (**e**,**f**) Spectrum of the extinction coefficient (*κ*_NC_) and refractive index of CsPbBr_3_ nanocrystals (*n*_NC_) are obtained using the two refractive indices of *n*_NC_(473 nm) = 2.085 ± 0.013 and *n*_NC_(632.8 nm) = 1.894 ± 0.011 in the Maxwell–Garnett approximation, where black solid line and green shadow represent the optimum average and error bar, respectively.

For a preliminary experiment, the refractive index of a cuvette (*n*_q_) was measured in the absence of a solution. In this case, the refractive index of air is used for *n*_sol_. Given the angle-dependent intensity in interference *I*(*θ*), the theoretical formula of Equation (1) was fitted. Two *n*_q_ at 473.0 nm and 632.8 nm were obtained to be 1.459 and 1.456, respectively. Subsequently, the cuvette was filled with CsPbBr_3_ NC solution, and *n*_sol_ was obtained with the same method. Regarding the combined optical effect of both NCs and toluene solvent, *n*_sol_ is specified using different terminology of the effective refractive index *n*_eff_. In Figure 3a, the concentration-dependent *n*_eff_ of the CsPbBr_3_ solution at 473 nm is plotted in terms of the volume fraction ($f_{v}=\nu N_{A}V_{NC}$), which can be estimated using the molar concentration of CsPbBr_3_ NCs (ν), Avogadro’s number (*N*_A_), and the average volume of NC (*V*_NC_). Likewise, *n*_eff_ of the CsPbBr_3_ solution measured at 632.8 nm is also shown in Figure 3b for increasing volume fraction. Due to the strong absorption at 473 nm, the decreased laser intensity deteriorates the measurement accuracy of *n*_eff_. It is noticeable that the measurement error bar at 473 nm (Figure 3a) becomes increased compared to *n*_eff_ at 632.8 nm (Figure 3b).

Provided that *n*_eff_ is measured at a selected wavelength, the refractive index of NCs (*n*_NC_) can be obtained as [48]:

(2) $$n_{NC}^{2}={-n}_{host}^{2}\frac{2\left(n_{eff}^{2}-n_{host}^{2}\right)+f_{v}(n_{eff}^{2}+2n_{host}^{2})}{\left(n_{eff}^{2}-n_{host}^{2}\right)-f_{v}(n_{eff}^{2}+2n_{host}^{2})},$$

where the refractive index of the host medium (toluene solvent) at 473 nm (*n*_host_ = 1.507) and 632.8 nm (*n*_host_ = 1.490) are also necessary. While *n*_eff_ of the CsPbBr_3_ solution shows a linear increase for volume fraction, a constant *n*_NC_ can be obtained. In other words, with the constant *n*_NC_, the theoretical *n*_eff_ can be described as:

(3) $$n_{eff}^{2}=n_{host}^{2}\frac{\left(n_{NC}^{2}+2n_{host}^{2}\right)+2f_{v}(n_{NC}^{2}-n_{host}^{2})}{\left(n_{NC}^{2}+{2n}_{host}^{2}\right)-f_{v}(n_{NC}^{2}-n_{host}^{2})}.$$

Therefore, *n*_NC_ can be obtained as a fitting parameter of Equation (3). This fitting method also provides a similar result compared to the method of Equation (2), which is averaging over the *n*_NC_ distribution for various volume fractions. In Figure 3a,b, the measured *n*_eff_ for volume fraction was compared with the Maxwell–Garnett model. The optimum *n*_NC_ at 473 nm (*n*_NC_ = 2.085 ± 0.013) and 632.8 nm (*n*_NC_ = 1.894 ± 0.011) were obtained, where the ratio of standard deviation to average refractive index ($\delta n_{NC}/{\bar{n}}_{NC}$) was found to be ~0.6%.

In Figure 3c, the spectrum of the effective absorption coefficient *α*_eff_(*λ*) is observed for various concentrations of CsPbBr_3_ NC solutions. We found that each *α*_eff_(*λ*) at a selected wavelength shows a linear increase with increasing concentration. This result indicates that the density of NCs increases uniformly without aggregation. Using the Kramers–Krönig relations, the effective refractive index spectrum *n*_eff_(*λ*) can also be obtained from *α*_eff_(*λ*) as:

(4) $$n_{eff}\left(\lambda \right)=n_{0}+\frac{P}{4\pi^{2}}\int_{0}^{\infty }\frac{\alpha_{eff}(\lambda^{\prime })}{1-{\left(\lambda /\lambda^{\prime }\right)}^{2}}d\lambda^{\prime },$$

where *P* denotes the Cauchy principal value of improper integration to avoid the singularity at *λ*′ *= λ*. Because a finite spectrum range of *α*_eff_(*λ*) is given for practical integration of Equation (4), the two references of *n*_eff_ at 473 nm and 632 nm were used to find the offset *n*_0_. In Figure 3d, the spectra of *n*_eff_(*λ*) were obtained for various concentrations with the two reference refractive indices.

As *n*_NC_ at a selected wavelength *λ* was obtained from the volume fraction (concentration) dependence of *n*_eff_ in Figure 3a,b, *n*_NC_(*λ*) spectrum can also be estimated from *n*_eff_(*λ*) spectrum. Additionally, this leads to the spectrum of extinction coefficient *κ*_NC_(*λ*) through the Kramers–Krönig relations as:

(5) $$\kappa_{NC}\left(\lambda \right)=\frac{P}{\pi }\int_{0}^{\infty }\frac{n_{NC}\left(\lambda^{\prime }\right)-n_{NC}\left(\infty \right)}{\lambda^{\prime 2}-\lambda^{2}}d\lambda^{\prime }.$$

Consequently, the imaginary and real spectrum of the complex refractive index ${\tilde{n}}_{NC}\left(\lambda \right)=n_{NC}\left(\lambda \right)+i\kappa_{NC}(\lambda )$ were obtained in Figure 3e and Figure 3f, respectively. Error bars (green shadow) were also shown around the average refractive indices (solid line). Regarding various measurements and analyses involved in estimating the complex refractive index of CsPbBr_3_ NC, the inaccuracy $\delta {\tilde{n}}_{NC}(\lambda )$ is quite natural. From the viewpoint of experiment, the angle-dependent interferometer provides n_eff_ in high precision (~0.6%), and the measurement accuracy of *α*_eff_(*λ*) is also fairly good. To obtain the molar concentration (*ν*), the formula of *A = ενd*_sol_ in the Beer-Lambert law was used. Given the molar extinction coefficient of toluene *ε* = 2.9 × 10^5^ M^−1^cm^−1^ and the light propagation length of solution *d*_sol_ = 10 mm, the molar concentration ν was estimated from measurable absorbance of A. For example, 1 mg/mL of CsPbBr_3_ NC solution dispersed in toluene leads to *ν* = 4.23 × 10^−6^ L mol^−1^.

Regarding the numerical integration process of the Kramers–Krönig relations in Equation (4), the limit of the finite spectrum seems to be compromised with the offset parameter n_0_, resulting in an excellent agreement between the modified *n*_eff_(*λ*) and the reference refractive indices measured at two different wavelengths (Figure 3d). However, the size inhomogeneity of NCs ($\delta L/\bar{L}$~22%) leads to a volume fraction error (*δf*) via a large volume inhomogeneity ($\delta V/\bar{V}$~65%). As a result, *δf* involved in Equations (2) and (3) also deteriorates the accuracy of *n*_NC_. While *n*_NC_(*λ*) is converted into *κ*_NC_(*λ*) through the Kramers–Krönig relations, this also propagates. Nevertheless, the real and imaginary spectrum of the complex refractive index of CsPbBr_3_ NC with the overall inaccuracy (<0.7%) can be quite a useful reference in optoelectronic device applications. While the intrinsic bromide vacancies may contribute to the measured refractive index [42], their effect is challenging to isolate due to the overwhelming influence of size-induced inhomogeneity in our colloidal system.

Furthermore, a comparison of our results with previous reports on the refractive index of CsPbBr_3_ nanocrystal film [42] and bulk film [62,63,64] measured with ellipsometry, which provides additional context for the significance of our approach. These studies reveal that the refractive index of CsPbBr_3_ is highly sensitive to morphological variations, influenced by factors such as substrate interactions, film thickness, and surface roughness. Our method, by directly measuring the refractive index of colloidal NCs in solution, avoids such morphological influences and aligns with the intrinsic properties of the NCs. Notably, our results demonstrate a similar range to the refractive indices observed for the film-state CsPbBr_3_ while accounting for the effect of morphological influence, suggesting that our approach may provide improved accuracy in applications where precise determination of the intrinsic optical constants is essential and morphological influences are minimal. This distinction highlights the utility of solution-state measurements as a complementary technique for studying nanoscale particles and emphasizes their relevance in applications where size uniformity and intrinsic material properties are important.

The solution-state method proposed in this study for measuring the intrinsic refractive index of CsPbBr_3_ NCs can also be employed to investigate quantum confinement effects. Here, the CsPbBr_3_ NCs used in this study exhibit a size that exceeds the exciton Bohr radius (~7 nm for CsPbBr_3_) [59,60,61], thereby diminishing the quantum confinement effects. In contrast, for smaller NCs, it is anticipated that quantum confinement will induce a blue shift at the absorption edge, accompanied by substantial shifts in the extinction coefficient and refractive index spectra. This provides a valuable opportunity to further explore the influence of quantum effects on the optical properties of nanocrystals by utilizing the approach introduced in this study.

## 4. Conclusions

We have addressed a systematic method to estimate the intrinsic complex refractive index spectrum from various concentrations of CsPbBr_3_ nanocrystal solution. The wavelength-specific effective refractive index of colloidal CsPbBr_3_ NCs was precisely measured with the Michelson interferometer, and the full spectrum of real and imaginary refractive index was obtained through the Kramers–Krönig relations. Using the effective refractive index of colloidal NCs, the full spectrum of the real and imaginary refractive index of intrinsic CsPbBr_3_ NCs was also established based on the Maxwell–Garnett effective medium approximation. This approach enables direct measurement of the intrinsic refractive index of nanocrystals in solution-state, overcoming the uncertainties associated with film-based measurements, such as those arising from thickness, surface roughness, and packing fraction of nanocrystals film. This method offers a robust framework for obtaining accurate refractive index spectra, which can be extended to a diverse range of quantum materials and nanoparticles. Such advancements provide a valuable framework for advancing optoelectronic device design and optimization.

## Data Availability

The data presented in this study are available on request from the corresponding author.
